# LSM2 is associated with a poor prognosis and promotes cell proliferation, migration, and invasion in skin cutaneous melanoma

**DOI:** 10.1186/s12920-023-01564-1

**Published:** 2023-06-13

**Authors:** Xiaofang Sun, Jianping Zhang, Jiayuan Hu, Qingdong Han, Zili Ge

**Affiliations:** 1 Department of Dermatology, the Second Affiliated Hospital of Jiaxing University, Jiaxing, Zhejiang China; 2 Department of Oral and Maxillofacial Surgery, the First Affiliated Hospital of Soochow University, Soochow University, Jiangsu, China

**Keywords:** SKCM, LSM2, Prognostic biomarker, Migration, Invasion

## Abstract

**Background:**

Skin cutaneous melanoma (SKCM) is an extremely malignant tumor that is associated with a poor prognosis. LSM2 has been found to be related to different types of tumors; however, its role in SKCM is poorly defined. We aimed to determine the value of LSM2 as a prognostic biomarker for SKCM.

**Methods:**

The expression profile of LSM2 mRNA was compared between tumor and normal tissues in public databases, such as TCGA, GEO, and BioGPS. LSM2 protein expression was explored using immunohistochemistry (IHC) on a tissue microarray containing 44 SKCM tissues and 8 normal samples collected at our center. Kaplan-Meier analysis was performed to assess the prognostic value of LSM2 expression in patients with SKCM. SKCM cell lines with LSM2 knockdown were used to determine the effects of LSM2. Cell counting kit-8 (CCK8) and colony formation assays were conducted to assess cell proliferation, whereas wound healing and transwell assays were carried out to assess the migration and invasion abilities of SKCM cells.

**Results:**

LSM2 was more highly expressed at the mRNA and protein levels in SKCM than that in normal skin. Moreover, elevated expression of LSM2 was associated with shorter survival time and early recurrence in patients with SKCM. The in vitro results revealed that the silencing of LSM2 in SKCM cells significantly inhibited cell proliferation, migration, and invasion.

**Conclusion:**

Overall, LSM2 contributes to malignant status and poor prognosis in patients with SKCM and may be identified as a novel prognostic biomarker and therapeutic target.

**Supplementary Information:**

The online version contains supplementary material available at 10.1186/s12920-023-01564-1.

## Background

Skin cutaneous melanoma (SKCM) is a highly aggressive type of skin cancer. Although SKCM accounts for a small fraction of dermatological cancers (< 10%), it is responsible for the highest number of skin cancer-related deaths worldwide [1]. Both hereditary and non-hereditary factors are involved in the development of SKCM [2, 3]. SKCM is distinguished by a wide range of heterogeneity in terms of histopathological presentation and clinical features [4, 5], genetic profiles [6, 7], and risk factors [8–10] (exposure to sun radiation, race, age, sex, number of nevi, family history, etc.). Accordingly, SKCM is among the most complex diseases and an important issue in the cancer field. With advances in genomics and clinical technology in recent decades, significant progress has been made in understanding SKCM biology and genetics, and therapeutic methods. However, owing to its continuously increasing incidence and lack of effective treatment for advanced stages, SKCM remains a major problem worldwide. Currently, the overall 5-year survival rate for patients with stage IV SKCM is only 19% [11]. Moreover, being a skin tumor, it is possible an early diagnosis and adequate surgical treatment, but nevertheless there are patients who present and advanced and metastatic melanoma at the diagnosis. Therefore, an urgent need exists to identify novel and efficient diagnostic and therapeutic biomarkers for SKCM and effective treatments for SKCM.

LSM2 belongs to the large “family of Sm-like” (LSM), which consists of 13 members (LSM1-LSM14B). Members of the LSM family are ubiquitous in nature, ranging from archaebacteria to humans. The LSM family has a highly conserved Sm domain called the Sm fold, which consists of an N-terminal α-helix, loop regions, and five strongly bent β-strands [12, 13]. The LSM family typically exists as a heptameric complex in vivo and participates in RNA-related functions. The LSM1-7 complex, which is located in the cytoplasm, interacts with decapping enzymes to make the mRNA sensitive to the 5′ to 3′ XRN-1 exonuclease [14]. The LSM2-8 complex, which is located in the nucleus, stabilizes U6 small nuclear RNA (snRNA) and promotes RNA decay [15]. Some members of the LSM family have been identified as oncologic genes in several tumors [16–18]. LSM2 was positively associated with ZNF76 overexpression, which predicts poor prognosis in patients with ovarian cancer (OV) [19]. Another study revealed that the promoter cytosine phosphate guanosine island (CGI) of LSM2 might be a novel candidate for OV hypomethylated tumor markers [20]. Genetic variants of LSM2, an mRNA splicing protein, were confirmed to be associated with lung cancer [21]. In our previous study, LSM2 overexpression was found to be associated with poor prognosis in patients with SKCM [22]. However, most prior studies were based on bioinformatic analyses. Accordingly, the role of LSM2 in SKCM requires further validation.

In this study, LSM2 mRNA expression was evaluated using public datasets. LSM2 protein expression was explored using immunohistochemistry (IHC), and its relationship with clinicopathological features was investigated. Furthermore, the effect of LSM2 on the biological behavior of the SKCM cell lines in vitro was determined. Overall, this study provides a basis for LSM2 as a poor prognostic biomarker and therapeutic target for SKCM.

## Methods

### Data and sample collection

We downloaded the RNA-seq data and corresponding clinical information of patients with SKCM (n = 469) from TCGA database (https://portal.gdc.cancer.gov/). In addition, data for 812 normal skin samples from the GTEx database were downloaded from UCSCXENA (https://xenabrowser.net/datapages/). The GSE15605 and GSE3189 mRNA expression data were downloaded from the Integrated Gene Expression Omnibus (GEO, https://www.ncbi.nlm.nih.gov/geo/). The GSE15605 dataset comprised 58 SKCM tissues and 16 normal skin tissues, whereas the GSE3189 dataset comprised 45 SKCM tissues and seven normal tissues. The BioGPS database (http://biogps.org/#goto=welcome) was used to explore the expression profile of LSM2 in cutaneous melanoma and normal skin cell lines.

A total of 44 SKCM tissues and 8 normal skin tissues were collected at our center between January 1, 2014 and January 1, 2017. In this study, the corresponding clinical and demographic data of 44 patients with SKCM who underwent radical surgery were collected, including age, sex, Clark level, Breslow depth, tumor tumor-nodule-metastasis (TNM) stage, and melanoma ulcer. All patients were followed-up until June 30, 2022. Disease-free survival (DFS) and overall survival (OS) were the endpoints of the study. All specimens were confirmed by two experienced pathologists. All human sample collections were approved by the Ethics Committee of our center (No. JEXY-ZFYJ076). This study adhered to the standards proposed by the Declaration of Helsinki, and written informed consent was obtained from all participants.

### Expression profile and prognostic value of LSM2 at the mRNA level

The RNA-seq of transcriptome information and corresponding clinicopathological data of patients with SKCM were downloaded from TCGAGDC (https://portal.gdc.cancer.gov/). The expression values of fragments per kilobase of gene per million fragments (FPKM) were converted to transcripts per kilobase of exon model per million mapped reads (TPM) for further analysis. The expression profile of LSM2 in TCGA and GTEx databases was explored using the Wilcoxon rank-sum test and visualized by “ggplot2” package in R software (p < 0.05 was set as statistic difference) (version 4.1.3). LSM2 expression in the GEO datasets and BioGPS database was analyzed and plotted using GraphPad Prism 5. The associations between LSM2 mRNA expression and OS and progression free survival (PFS) in TCGA dataset were assessed using the “survival” and “survminer” packages. The log-rank test was used to analyze survival in the different groups. The time receiver operator characteristic (ROC) curves at 1, 3-, and 5-years were used to assess the predictive accuracy of LSM2 mRNA. For the Kaplan-Meier (KM) curves, p-value and hazard ratios (HR) with 95% confidence intervals (CI) were obtained using the log-rank test and univariate Cox regression. An HR > 1 indicates that the gene is a risk factor, and an HR < 1 indicates that the gene is a protective factor. The median time indicates the time corresponding to the survival rate of the high- and low-expression groups at 50% (i.e., median survival time).

### Gene function in the cell lines

We determined whether LSM2 is essential for cutaneous melanoma cells using the Cancer Dependency Map [23, 24] (DepMap, https://depmap.org/portal/interactive/) dataset. The DepMap is a user-friendly website for large-scale multiomics screening programs, including the Cancer Cell Line Encyclopedia (CCLE), Expression 22Q2 Public, CRISPR-Cas9 (DepMap 22Q2 Public + Score, Chronos), and RNAi (Achilles + DRIVE + Marcote, DEMETER2). Based on the computational algorithm model in DepMap, the gene effect scores of genes in the RNAi screening dataset and CRISPR-Cas9 knockout screens were obtained. These two scores can be used to determine the effect of knocking down or knocking out a gene [24, 25]. A negative score indicates slower cell line growth, whereas a positive score represents faster cell line growth after the experimental operation. Generally, the cut-off is set as -0.5, which indicates significant depletion in a cell line, whereas − 1 indicates strong killing [26]. Herein, the cut-off was set at -0.5 based on the DepMap website. The dependency on LSM2 was explored using eight melanoma cell lines (including A2058, A375, MeWo, SKMEL-2, SKMEL-28, SKMEL-24, SKMEL-30, and SKMEL-1) in the Expression 22Q2 Public, CRISP, and RNAi datasets by the “Data Explore” module in Depmap.

### Tissue microarray (TMA) and IHC analysis

TMA is a method in which many small disks of samples are gathered from standard histologic specimens and placed on recipient paraffin, enabling the convenient and simultaneous analysis of hundreds of cases. Hematoxylin and eosin (HE)-stained slides were re-observed, and the most representative slide in the tumor field was selected. Normal tissue samples were selected in the same manner. In the formalin-fixed and paraffin-embedded blocks, 3 mm cores were collected in the most representative tumor field and transferred to an empty paraffin block for TMA blocking. In this study, the TMA included 88 cores from tumor tissues and eight cores from normal skin samples. Each tumor sample had two cores, whereas each normal tissue sample had one core.

TMA was baked at 59 ℃ in an incubator for 60 min before dewaxing and rehydration. Thereafter, the TMA was incubated with anti-LSM2 antibody (Lifespan, US, https://www.lsbio.com/antibodies/ihc-plus-lsm2-antibody-snrnp-antibody-clone-at2b2-elisa-ihc-wb-western-ls-b8972/194131) at 1:100 dilution at 4 °C overnight, followed by the secondary antibody for 30 min at room temperature. The immunohistochemical staining assessments were interpreted by two experienced pathologists who were blinded to the clinical and pathological data. The extent and intensity of staining were recorded. The staining extent scores of LSM2 were 0, 1, 2, 3, and 4, which represented 0, 1–25%, 26–50%, 51–75%, and 76–100%, respectively. The intensity staining scores of LSM2 were 0, 1, 2, and 3, which represented negative, weak, moderate, and strong staining, respectively. The final expression score of LSM2 protein in the specimen was multiplied by the staining score and the intensity staining score. According to the final score, samples were divided into low and high with the median cut off.

### Cell culture and transfection

The human malignant melanoma cell lines, A2058, A375, MeWo, SK-MEL-2, and SK-MEL-28, and the normal skin cell line, HEMa-LP, were purchased from Pricella (Wuhan, Hubei, China). STR matching analysis was used to confirm all cell lines. All cells were cultured at 37 °C with 95% humidity and 5% CO_2_.

The gene sequence of LSM2 was obtained from the National Center for Biotechnology Information (NCBI) database (https://www.ncbi.nlm.nih.gov/). Three small interfering RNA (siRNAs) were designed and synthesized based on the LSM2 gene sequence by Tsingke Biotechnology (Beijing, China): siLSM2-1 5’-CCAUUCUGUGGAUCAGUAU-3,’ siLSM2-2 5’-CUCACAUGUUAUCAGUGAA-3,’ and siLSM2-3 5’- CCAGCAGAUGAGGUCGACA − 3.’ To silence the LSM2 gene, melanoma cell lines were transfected with siRNA using a Transfection Reagent (INTERFERin, French).

### RNA extraction and real-time polymerase chain reaction (RT-PCR)

Total RNA was isolated from the cell lines using TRIzol Reagent (Haoke Biotechnology, Hangzhou, China), according to the manufacturer’s protocol. cDNA was synthesized with All-in-One First-Strand Synthesis MasterMix (AboRo, Shenzhen, China), according to the manufacturer’s protocol, using 1 µg of total RNA. The mRNA level of LSM2 was determined via RT-PCR with Taq SYBR Green qPCR Premix (AboRo, Shenzhen, China). The following primer sequences were used: LSM2, forward 5’-ATTCTGTGGATCAGTATCTC-3’ and reverse 5’-TCACTGTTTCTGCTGCAGGG-3’; GAPDH, forward 5’-TCAAGAAGGTGGTGAAGCAGG-3’ and reverse 5’-TCAAAGGTGGAGGAGTGGGT-3’. All samples were run in triplicate. GAPDH was used as an internal control.

### Western blot analysis

Total protein was isolated and quantified using the BCA Protein Assay Kit (Sangon Biotech, Shanghai, China). Twenty micrograms of protein per lane was separated via electrophoresis on a 10% SDS-PAGE gel and transferred onto a PVDF membrane. The membrane was blocked with TBST containing 5% skim milk for 1 h at room temperature and incubated with the anti-LSM2 antibody (Lifespan, USA, https://www.lsbio.com/antibodies/ihc-plus-lsm2-antibody-snrnp-antibody-clone-at2b2-elisa-ihc-wb-western-ls-b8972/194131) at 1:1000 dilution at 4 °C overnight. Thereafter, the membrane was incubated with horseradish peroxidase-conjugated sheep anti-mouse secondary antibody (GAPDH, Proteintech, USA) for 1 h at room temperature. The bands were detected using an ECL system (Millipore, MA, USA).

### Cell counting Kit-8 assay and colony formation assays

The cell proliferation ability of melanoma cells was assessed using the Cell Counting Kit-8 kit (CCK-8, APExBIO, USA). A total of 3 × 10^3^ cells per well were inoculated into 96-well plates at 24 h after transfection. CCK-8 reagent was added to each well after incubation of the cells at 37 °C for 0, 24, 48, 72 h, respectively. The absorbance of each well was measured at a wavelength of 450 nm. In terms of the cell colony formation assay, 5 × 10^2^ melanoma cells were cultured in 6-well plates for 10 days at 37 °C and 5% CO_2_. The cells were then stained with 0.1% crystal violet for 15 min and counted using the ImageJ software. Each experiment consisted of three independent tests.

### Wound healing assay

For the wound healing assay, 2 × 10^5^cells were seeded per well in a 6-well plate. Melanoma cells were cultured after LSM2 transfection with either siLSM2 or negative control at 37 °C and 5% CO_2_. Wounds were generated by scratching the monolayer of cells with a 100-ul pipette tip, and a photograph of the wounded field was captured at 0, 24, and 48 h. The scratch area was measured by ImageJ. The migration rate was quantified by measuring the ration of the wound closure area between 0 and 24 h. The results are displayed as % migration area. The difference among Blank, NC, and siLSM2 groups were analyzed by ANOVA method. Each experiment consisted of three independent tests.

### Transwell migration and invasion assays

A trans-well compartment with 8 μm pores was used (R&D Systems, USA). For the migration assay, 5 × 10^4^ cells were inoculated in the upper chambers (serum-free media). A total of 800 µl of serum-containing medium was added to the lower chamber. For the invasion assay, a Transwell chamber was coated with Matrigel (R&D Systems, USA), and 5 × 10^4^ cells were added to the top of the coated filters. After the cells were incubated for 24 h in a cell culture incubator, they were washed twice with PBS, fixed with methanol for 30 min, and stained with 0.1% crystal violet for 15 min. All cells were captured, and three high-power areas were randomly selected for cell counting.

### Statistical analysis

The data obtained from our center were analyzed using SPSS 20. LSM2 protein expression levels among the different groups and the relationships between clinicopathological features were analyzed using the χ^2^ test or Fisher’s exact χ^2^ test. The association between LSM2 protein expression and OS and DFS was evaluated using KM survival analysis. To further explore the independent risk factors for the clinical data, univariate and multivariate Cox regression analyses were conducted. The in vitro experiments in this study were all independent replicated three times. The ImageJ software was used to count cell number and quantify scratch area. For the comparison of multiple groups of data, ANOVA method was used and t-tests were used on continuous variables to clarify the comparison between groups. P < 0.05 was considered to indicate statistical significance.

## Results

### Expression profile of LSM2 at the mRNA level

**Fig. 1:**
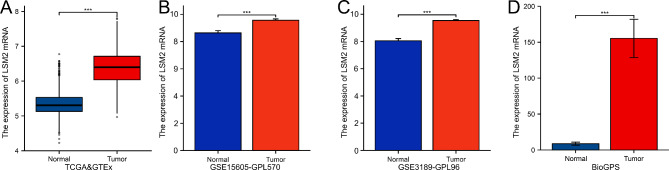
LSM2 is highly expressed in SKCM in the bioinformatics databases **A**. LSM2 mRNA was significantly overexpressed in SKCM tissues compared with normal skin tissues in TCGA and GTEx datasets **B, C**. In the GSE15605 and GSE3189 datasets, LSM2 expression in SKCM tissues was significantly higher than that in normal skin tissues **D**. The expression of LSM2 in the melanoma cell lines was higher than that in the normal cell line according to the BioGPS database. * P ≤ 0.05; **P ≤ 0.01; ***P ≤ 0.001

In TCGA and GTEx databases, the LSM2 mRNA levels were significantly higher in SKCM than in normal skin tissues (*p* < 0.001, Fig. 1A). The GSE15605 (*p* < 0.001, Fig. 1B) and GSE3189 (*p* < 0.001, Fig. 1C) results also confirmed that LSM2 mRNA was markedly elevated in SKCM tissues compared with normal skin tissues. The expression profile of LSM2 in melanoma cell lines and normal skin was explored using the BioGPS database, and LSM2 was found to be expressed at elevated levels in cutaneous melanoma cell lines (*p* < 0.001, Fig. 1D).

### Prognostic values of LSM2 in SKCM at the mRNA level based on TCGA

**Fig. 2:**
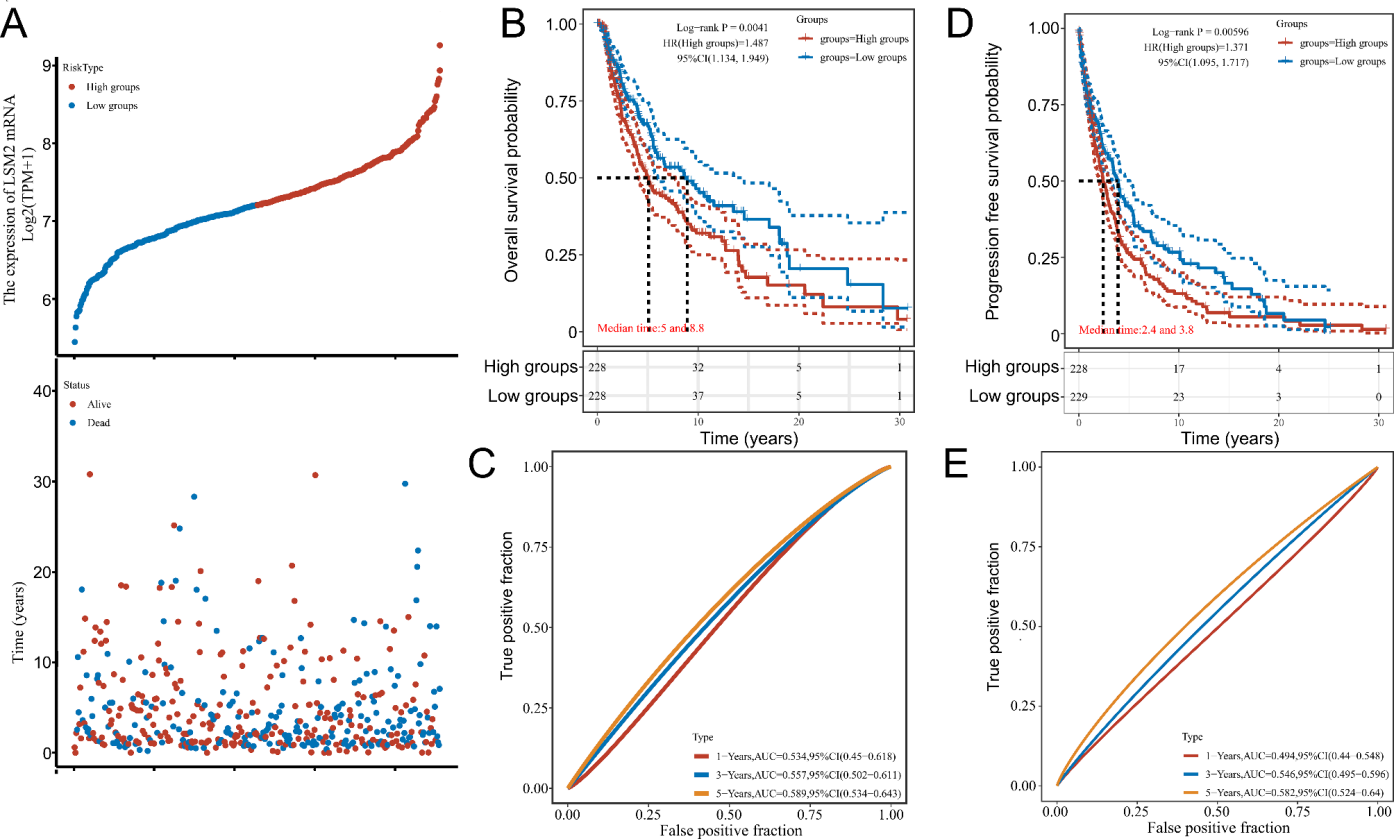
LSM2 mRNA expression is associated with the OS and PFS of patients with SKCM in TCGA dataset (**A**) LSM2 Expression Profile, Survival Status, and Survival Time were analyzed and visualized by “ggrisk” package. The top scatterplot indicates LSM2 expression (TPM) from low to high. Red represents the LSM2 high expression group while blue represents the LSM2 low expression group. The scatter plot distribution displays the survival status and survival time. (**B**) Prognostic value of LSM2 in OS. **C**. Time-dependent ROC of LSM2 for predicting OS. **D**. Prognostic value of LSM2 in PFS. **E**. Time dependent ROC of LSM2 for predicting PFS. **B** and **D** were performed by “survival” and “survminer” package. **C** and **E** were analyzed by “timerROC”package.

According to the median LSM2 expression value, patients with SKCM were split into high and low LSM2 expression groups. The LSM2 expression profile, survival status, and survival time in TCGA dataset are displayed using scatterplots and heatmaps (Fig. 2A). High LSM2 expression was significantly associated with short OS (median time: 5 versus 8.8 years) (log-rank *p* = 0.0041, HR (High group) = 1.487, 95%CI (1.134, 1.949)) (Fig. 2B), and time-dependent ROC curves indicated that LSM2 had moderate specificity and sensitivity for predicting OS (1 − Years, AUC = 0.534, 95%CI (0.45 − 0.618); 3 − Years, AUC = 0.557, 95%CI (0.502 − 0.611); 5 − Years, AUC = 0.589, 95%CI (0.534 − 0.643)) (Fig. 2C). High expression of LSM2 was associated with decreased PFS (median time: 2.4 versus 3.8 years) (log-rank *p* = 0.00596, HR (high group) = 1.371, 95%CI (1.095, 1.717)) (Fig. 2D). The time-dependent accuracy of LSM2 expression in predicting PFS at 1, 3-, and 5-years was explored via time-dependent ROC analysis (1 − Years, AUC = 0.494, 95%CI (0.44 − 0.548); 3 − Years, AUC = 0.546, 95%CI (0.495 − 0.596); 5 − Years, AUC = 0.582, 95%CI (0.524 − 0.64)) (Fig. 2E).

### CRISPR-Cas9 and RNAi of LSM2 in melanoma cell lines using DepMap

**Fig. 3:**
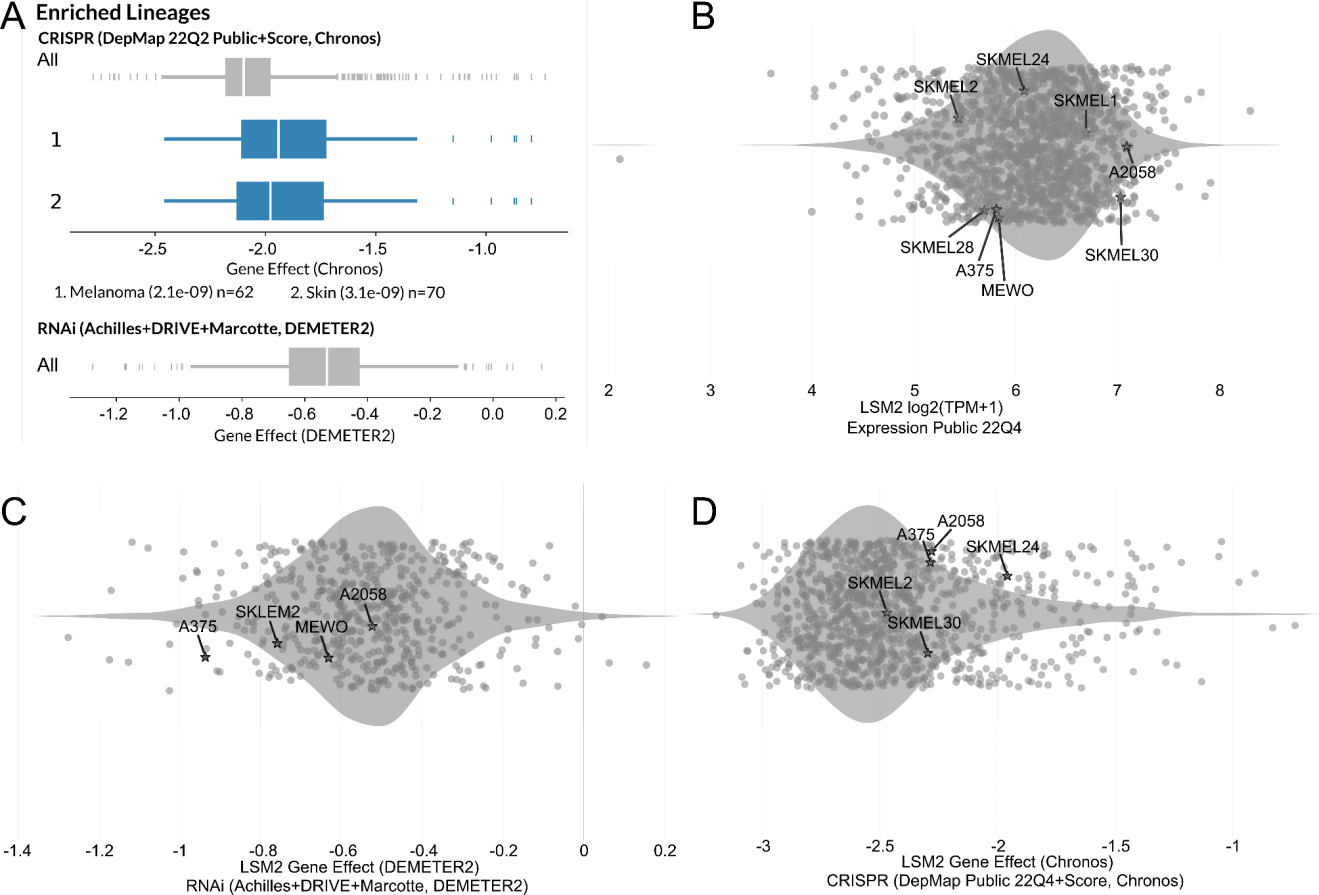
DepMap screening of the LSM2 gene **A**. Enriched lineages of LSM2. Enriched lineages have p-values < 0.0005 (shown in parentheses). N = 62 indicates the number of cell lines plotted in melanoma lineage. N = 70 indicates the number of cell lines plotted in skin lineage **B**. In LSM2, the gene effect scores for eight melanoma cell lines in the Expression 22Q2 public dataset. The gene effect score of LSM2 in the A2058 (7.0911), A375 (5.8084), MeWo (5.8329), SKMEL-2 (5.4349), SKMEL-28 (5.6633), SKMEL-24 (6.0824), SKMEL-30 (7.0297), and SKMEL-1(6.6871) cell lines were positive **C**. RNAi screening of LSM2 in the melanoma cell lines. In LSM2, the A2058 (-0.5231), A375 (-0.9363), MeWo (-0.6314), and SKMEL-2 (-0.7581) cell lines had a score below-0.5 for the RNAi data **D**. CRISPR-Cas9 screening of LSM2 in the melanoma cell lines. In the CRISP dataset, LSM2 gene effect scores for A2058 (-2.0791), A375 (-1.8848), SKMEL-2 (-2.1033), SKMEL-24 (-1.9768), and SKMEL-30 (-1.6849) cell lines were below the cut-off value (-0.5)

We evaluated the gene effect scores of LSM2 in melanoma cell lines using “Data Explore” module in DepMap. The enriched lineages of LSM2 were melanoma and skin cell lines (Fig. 3A). In the Expression 22Q2 Public dataset, the gene effect score of LSM2 in the A2058 (7.0911), A375 (5.8084), MeWo (5.8329), SKMEL-2 (5.4349), SKMEL-28 (5.6633), SKMEL-24 (6.0824), SKMEL-30 (7.0297), and SKMEL-1(6.6871) cell lines were positive (Fig. 3B). RNAi data were not found for the MeWo, SKMEL-28, SKMEL-24, and SKMEL-1 cell lines. In LSM2, the A2058 (-0.5231), A375 (-0.9363), MeWo (-0.6314), and SKMEL-2 (-0.7581) cell lines had a score below the cut-off value (-0.5) of the gene effect score for the RNAi data (Fig. 3C). In the CRISP dataset, LSM2 gene effect scores for the MeWo, SKMEL-28, and SKMEL-1 cell lines were not available, whereas those for the A2058 (-2.0791), A375 (-1.8848), SKMEL-2 (-2.1033), SKMEL-24 (-1.9768), and SKMEL-30 (-1.6849) cell lines were below the cut-off value (-0.5) of the LSM2 gene effect (Fig. 3D).

### LSM2 protein expression patterns and clinicopathological parameters of the clinical samples

**Fig. 4:**
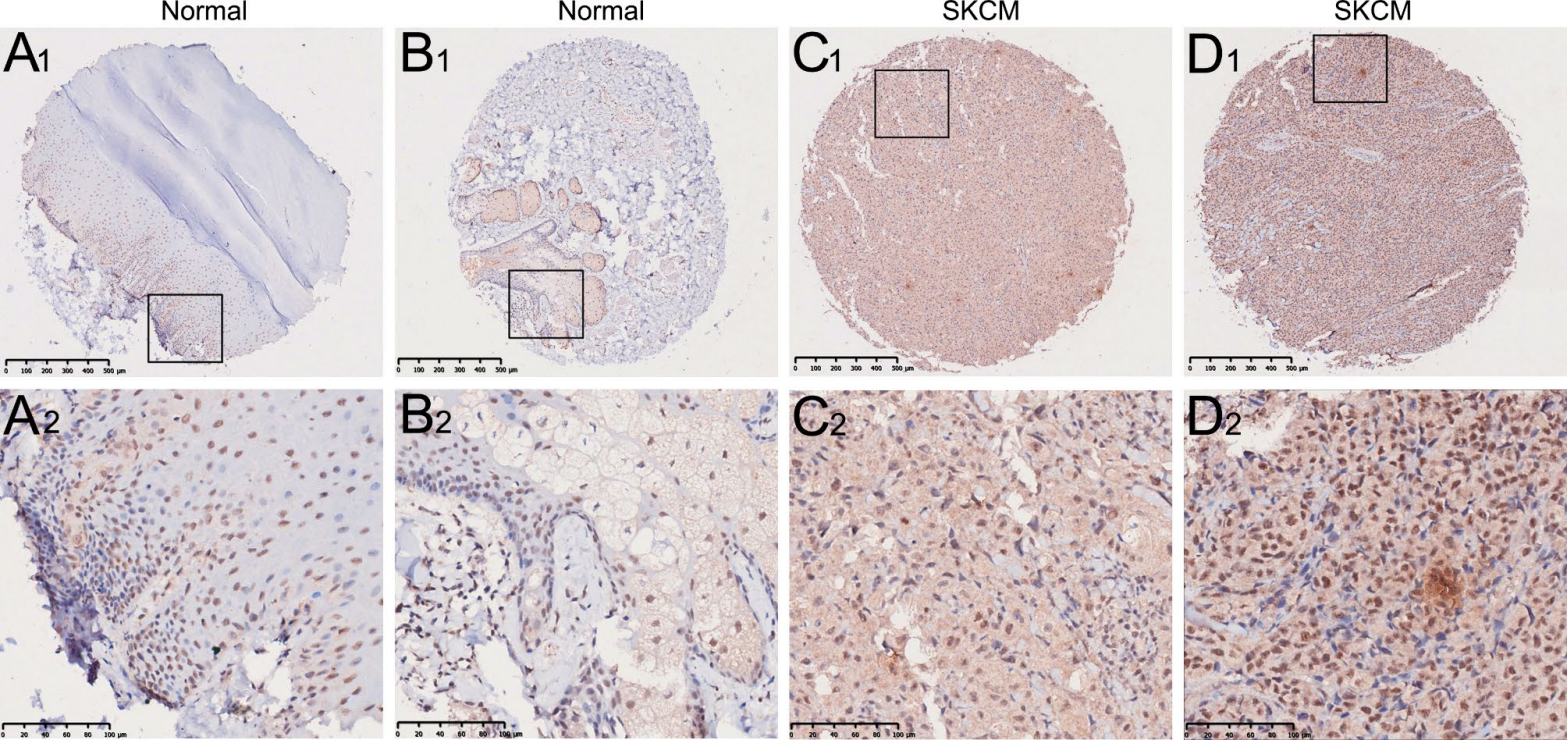
LSM2 protein expression in SKCM tissues and normal skin tissues The LSM2 protein displayed general nuclear and cytoplasmic expression in cells. Representative images of different staining of LSM2 (A_1 − 2_-D_1 − 2_). A_1 − 2_, B_1 − 2_. weak intensity of LSM2 in normal skin tissues; C_1 − 2_. weak intensity of LSM2 in SKCM tissues; D_1 − 2_. moderate intensity of LSM2 in SKCM tissues

Considering the expression profile of LSM2 mRNA in bioinformatics databases, we explored LSM2 protein expression in 44 normal SKCM tissues and eight normal skin tissues using TMA-based IHC. Based on IHC, LSM2 was mainly located in the nucleus and cytoplasm of SKCM cells. The different staining patterns of LSM2 are shown in Fig. 4. LSM2 protein levels were higher in SKCM tissues than in normal skin tissues (Fig. 4 A_1 − 2_, B_1 − 2_, C_1 − 2_, and D_1 − 2_). Of the 44 SKCM samples, 31 (72.73%) were moderately stained, while 13 (29.54%) were weakly stained. All 8 normal skin specimens displayed weak staining. Based on the median LSM2 protein expression value, patients with SKCM were split into high and low LSM2 expression groups to further identify the correlations between LSM2 expression and clinical features. The analysis revealed that high LSM2 expression was significantly associated with melanoma ulcers (*p* = 0.002), T stage (*p* = 0.003), N stage (*p* = 0.035), M stage (*p* = 0.046), and Clark level (*p* = 0.012) (Table 1).

**Table 1:** Correlation between LSM2 protein expression and clinicopathological features

Variables	Total(n = 44)	LSM2 protein expression		P value	
		High(n = 22)	Low(n = 22)		
Sex					
Male	21	10	11	0.763	
Female	23	12	11		
**Age, years**					
≤ 60	25	12	13	0.761	
>60	19	10	9		
**Ulcer**					
Yes	19	15	4	0.002	
No	25	7	18		
**T stage**					
T_1_-T_2_	14	2	12	0.003	
T_3_-T_4_	30	20	10		
**N stage**					
N_0_-N_1_	21	7	14	0.035	
N_2_-N_3_	23	15	8		
**M stage**					
M_0_	36	15	21	0.046	
M_1_	8	7	1		
**Clark level**					
I- III	27	9	18	0.012	
IV-V	17	13	4		
**Breslow depth**					
≤ 3 mm	18	5	13	0.031	
>3 mm	26	17	9		

### Prognostic value of LSM2 in clinical samples at the protein level

**Fig. 5:**
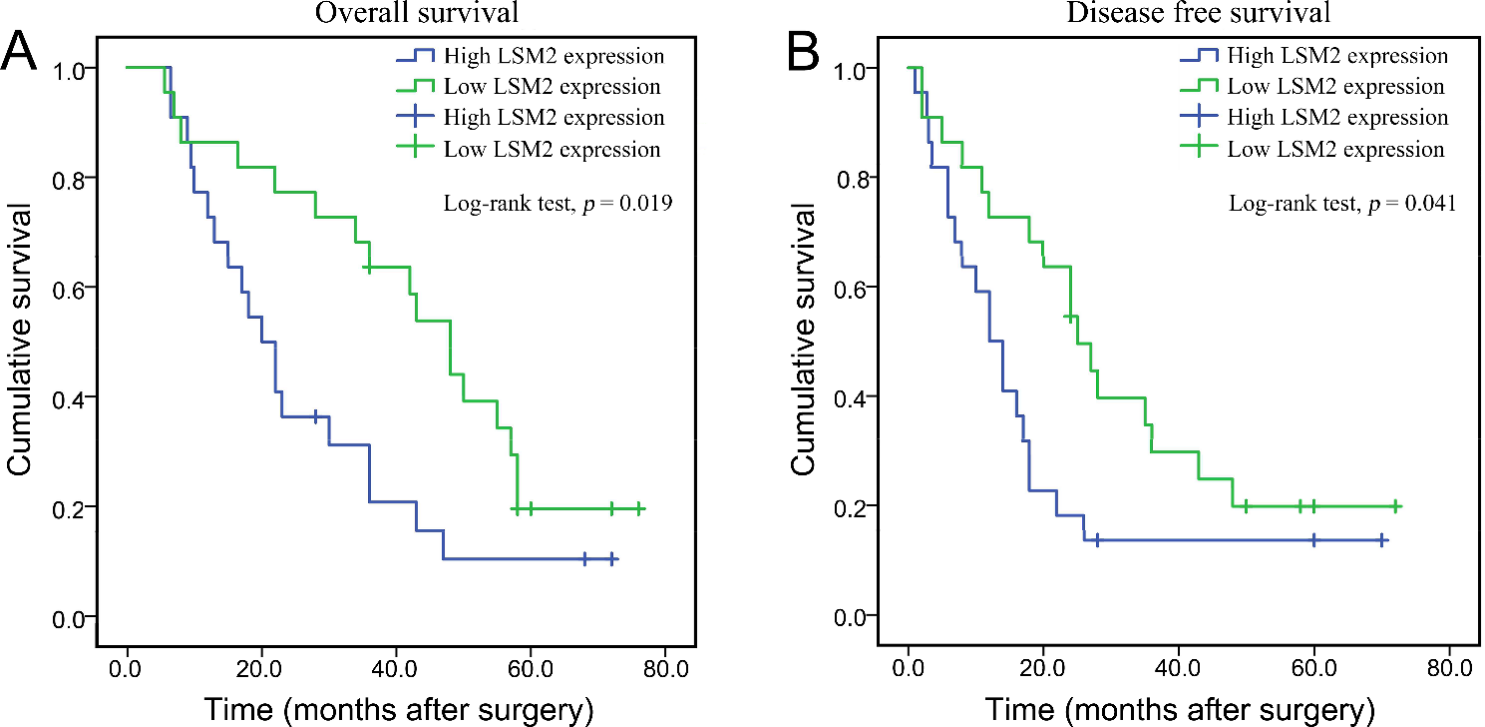
The prognostic value of the LSM2 protein in patients with SKCM in clinical cohort **A**. High expression of LSM2 protein indicated poor OS. **B**. High expression of LSM2 protein was related to early DFS.

To better understand the relationship between LSM2 protein and SKCM, a KM plot was constructed. High expression of LSM2 protein was associated with poor OS (median OS 20 vs. 48 months, *p* = 0.019, Fig. 5A) and early DFS (median DFS 12 vs. 25 months, *p* = 0.041, Fig. 5B) in patients with SKCM. Univariate analysis revealed that LSM2 expression (*p* = 0.002), pathological stage (*p* = 0.006), T stage (*p* = 0.032), N stage (*p* = 0.042), M stage (*p* < 0.001), ulceration (*p* = 0.006), Clark level (*p* = 0.001), and Breslow depth (*p* = 0.007) play important roles in the prognosis of SKCM (Table 2). Multivariate analysis further confirmed that pathological stage (*p* = 0.012), Clark level (*p* = 0.002), and Breslow depth (*p* = 0.003) are independent prognostic factors for patients with SKCM, while ulceration (*p* = 0.460) and LSM2 expression (*p* = 0.107) are not independent prognostic factors (Table 3).

**Table 2:** Univariate analyses of the clinicopathological characteristics associated with OS.

Variables		OS	
		Hazard ratio (95% CI)	*P* Values
Pathological stage(III–IV vs. I–II )		2.053(1.229–3.341)	0.006
T stage (T3-T4 vs. T1-T2)		2.246 (1.072–4.706)	0.032
N stage (N3-N4vs.N0-N1)		1.973 (1.026–3.795)	0.042
M stage (M1 vs. M0)		17.870 (5.597–57.051)	<0.001
Age, year ( ≧ 60vs.<60)		0.955 (0.500-1.824)	0.889
Sex (Female vs. Male)		1.262 (0.658–2.422)	0.484
Ulceration (No vs. Yes)		0.399 (0.207–0.768)	0.006
Clark level (I&II&III vs. IV&V)		0.304 (0.155–0.594)	0.001
Breslow depth (> 3 vs.≤3)		2.643 (1.310–5.334)	0.007
LSM2 expression (Low vs. High)		0.340 (0.171–0.677)	0.002

**Table 3:** Multivariate analyses of factors associated with OS.

Variables		OS	
		Hazard ratio (95% CI)	*P* Values
Pathological stage(III–IV vs. I–II )		1.925 (1.157–3.204)	0.012
Ulceration (No vs. Yes)		1.398 (0.575–3.404)	0.460
Clark level (I&II&III vs. IV&V)		0.269(0.119–0.607)	0.002
Breslow depth (>3 vs.≤3)		3.291 (1.484–7.297)	0.003
LSM2 expression (Low vs. High)		0.485 (0.201–1.169)	0.107

### Expression profile of LSM2 in the SKCM cell lines and transfection

**Fig. 6:**
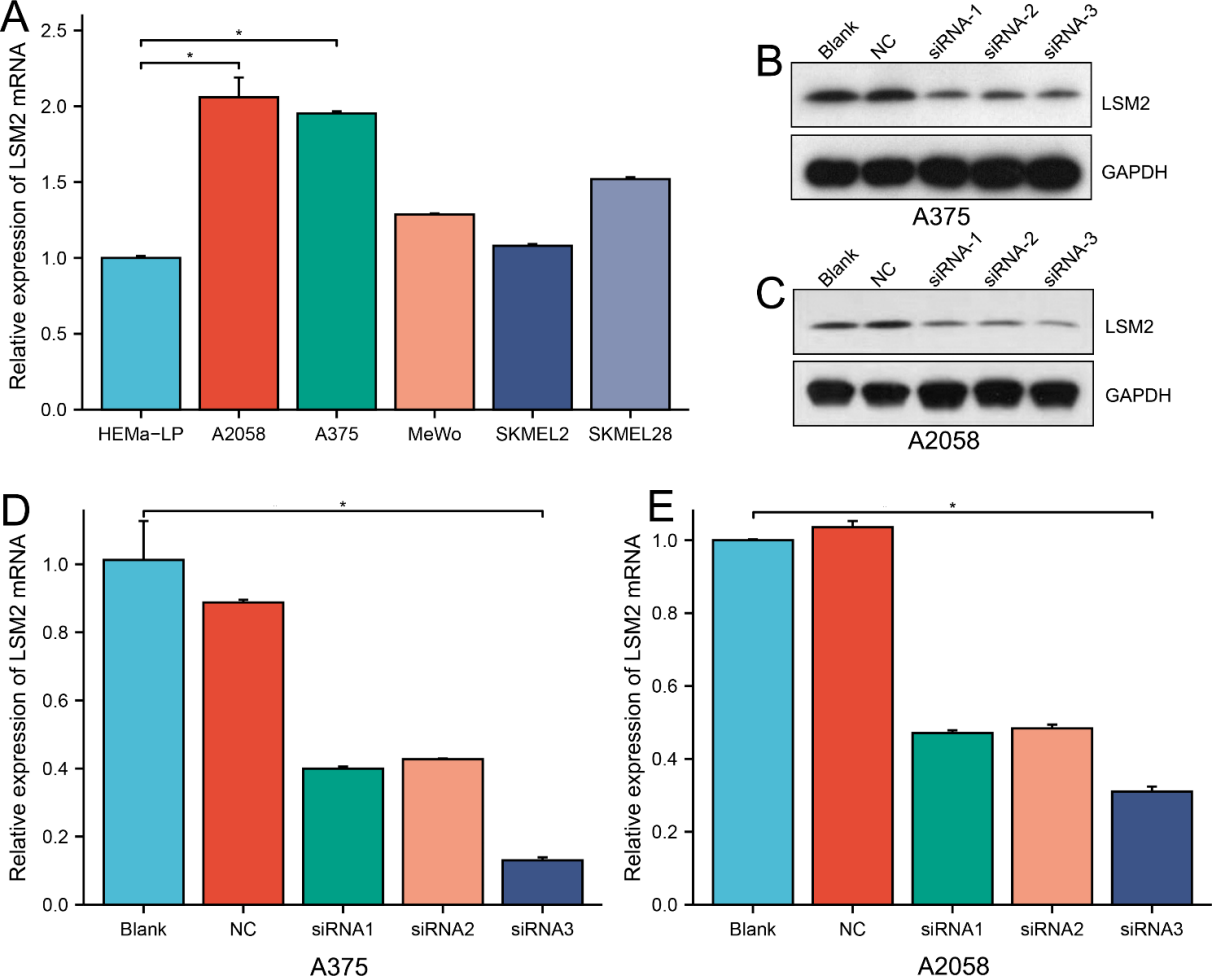
LSM2 expression profile and transfection in cell lines **A**. Relative LSM2 expression was investigated in five cutaneous melanoma cell lines and compared with that in the normal human epidermal melanocyte cell line, HEMa-LP. LSM2 expression was explored using RT-PCR and normalized to GAPDH expression **B, C**. Western blot analysis of LSM2 protein expression in A375 or A2058 cells, negative control cells, and cells transfected with siRNA-1, siRNA-2, and siRNA-3. **D, E**. LSM2 was silenced by siRNA-1, siRNA-2, and siRNA-3 in A375 or A2058 cells. LSM2 expression was examined using RT-PCR.

The expression of LSM2 was assessed in the normal skin cell line (HEMa-LP) and cutaneous melanoma cell lines (A2058, A375, MeWo, SKMEL-2, and SKMEL-28) using RT-PCR. LSM2 expression was found to be significantly enhanced in the A375 and A2058 cell lines compared to that in the normal human epidermal melanocyte cell line, HEMa-LP (Fig. 6A, p < 0.05). As the expression of LSM2 was especially high in the A375 and A2058 cell lines, we opted to perform subsequent experiments using these two cell lines. LSM2 expression was reduced in the siRNA-1, siRNA-2, and siRNA-3 groups compared with the negative control (NC) group or blank group. Further, siRNA-3 displayed a slightly better knockdown efficiency, which was verified by WB (Fig. 6B, C) and RT-PCR (Fig. 6D, E). The original data of Fig. 6B C were presented as Supplementary Figure S1 and Supplementary Figure S2.

### Knockdown of LSM2 inhibits SKCM cell proliferation, migration, and invasion

**Fig. 7:**
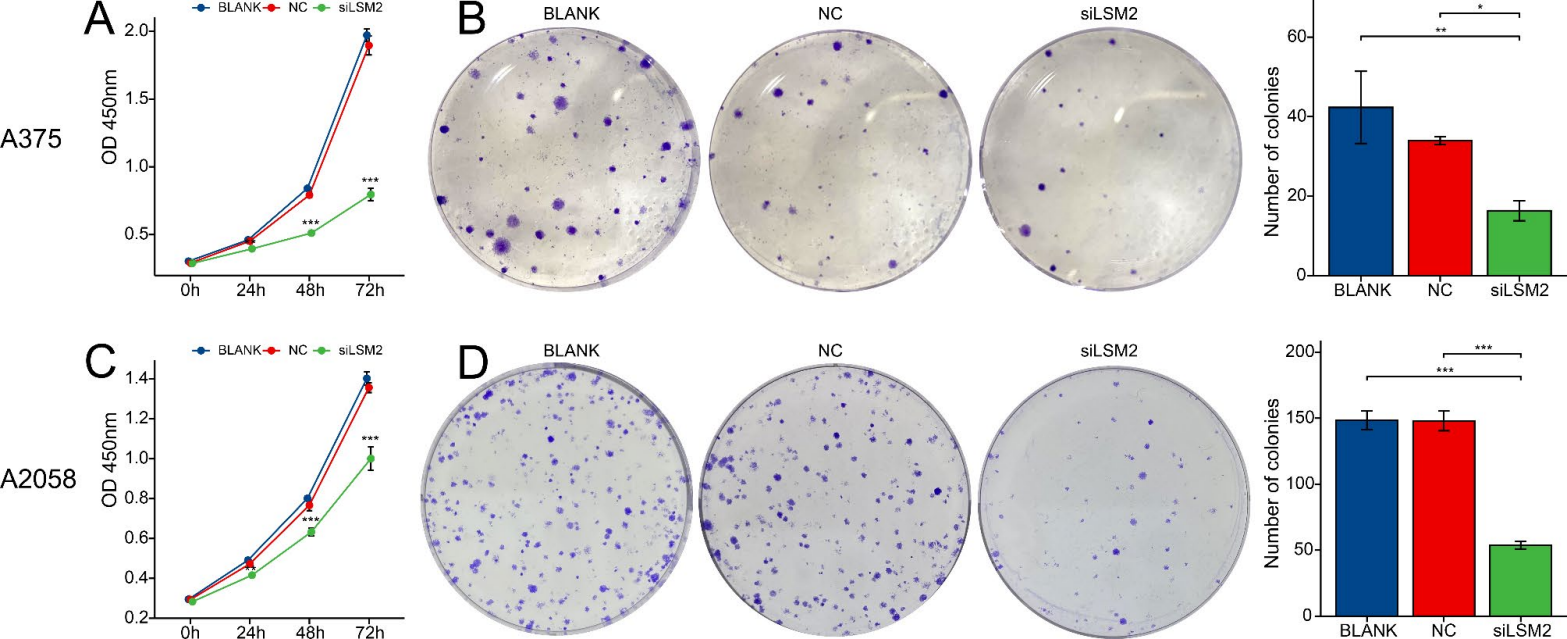
Knockdown of LSM2 inhibits cell proliferation **A, C**. Cell proliferation of A375 and A2058 cells was explored using CCK-8 assays **B, D**. Colony formation assay using A375 or A2058 cells revealed a significant decrease in the colony formation of cells with LSM2 knockdown

**Fig. 8:**
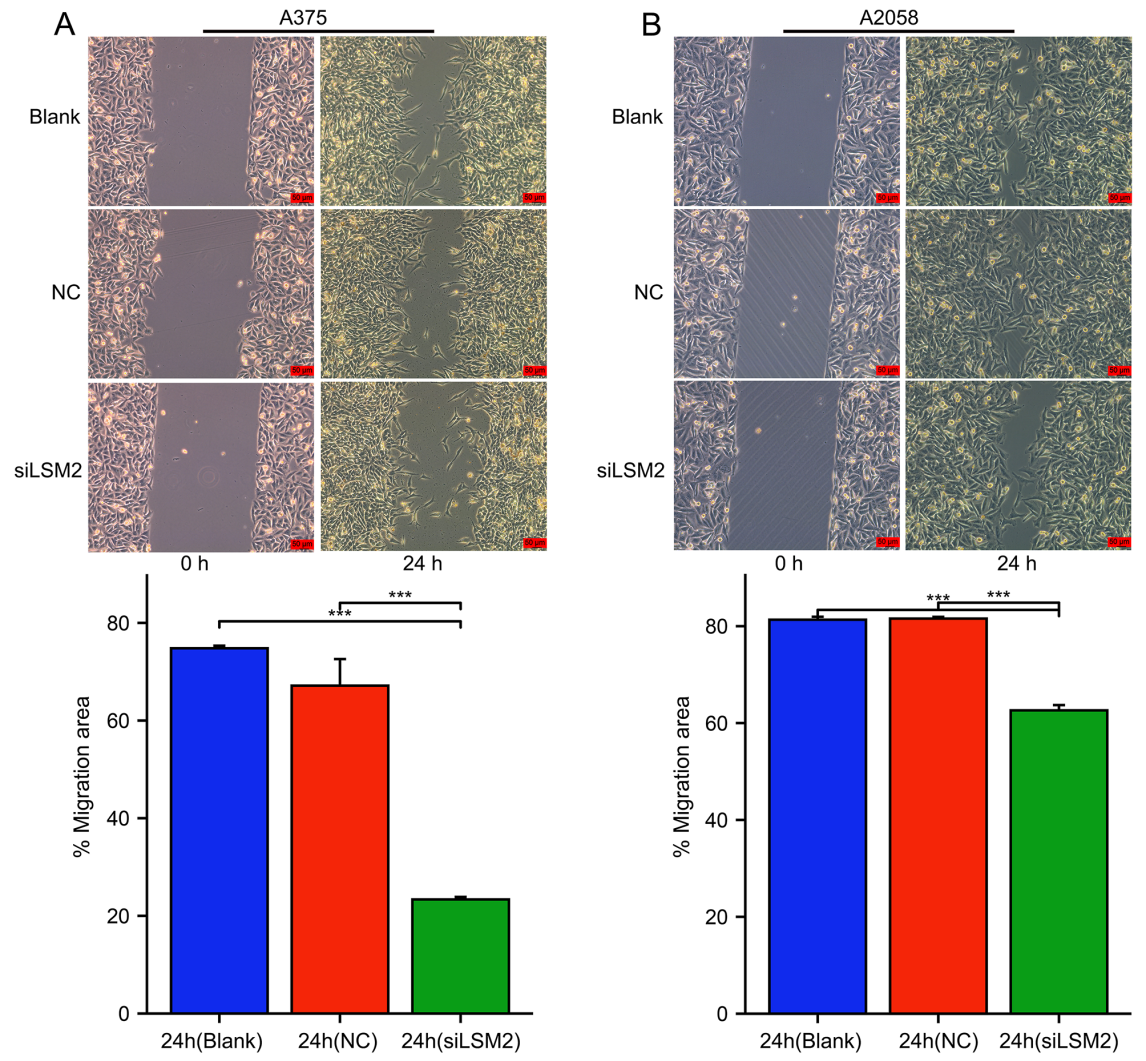
The effect of LSM2 knockdown inhibits the migration of SKCM cells **A, B** The representative images of wound healing assays in A375 and A2058 cell lines. The migration rate of A375 and A2058 were measured by % migration area

**Fig. 9:**
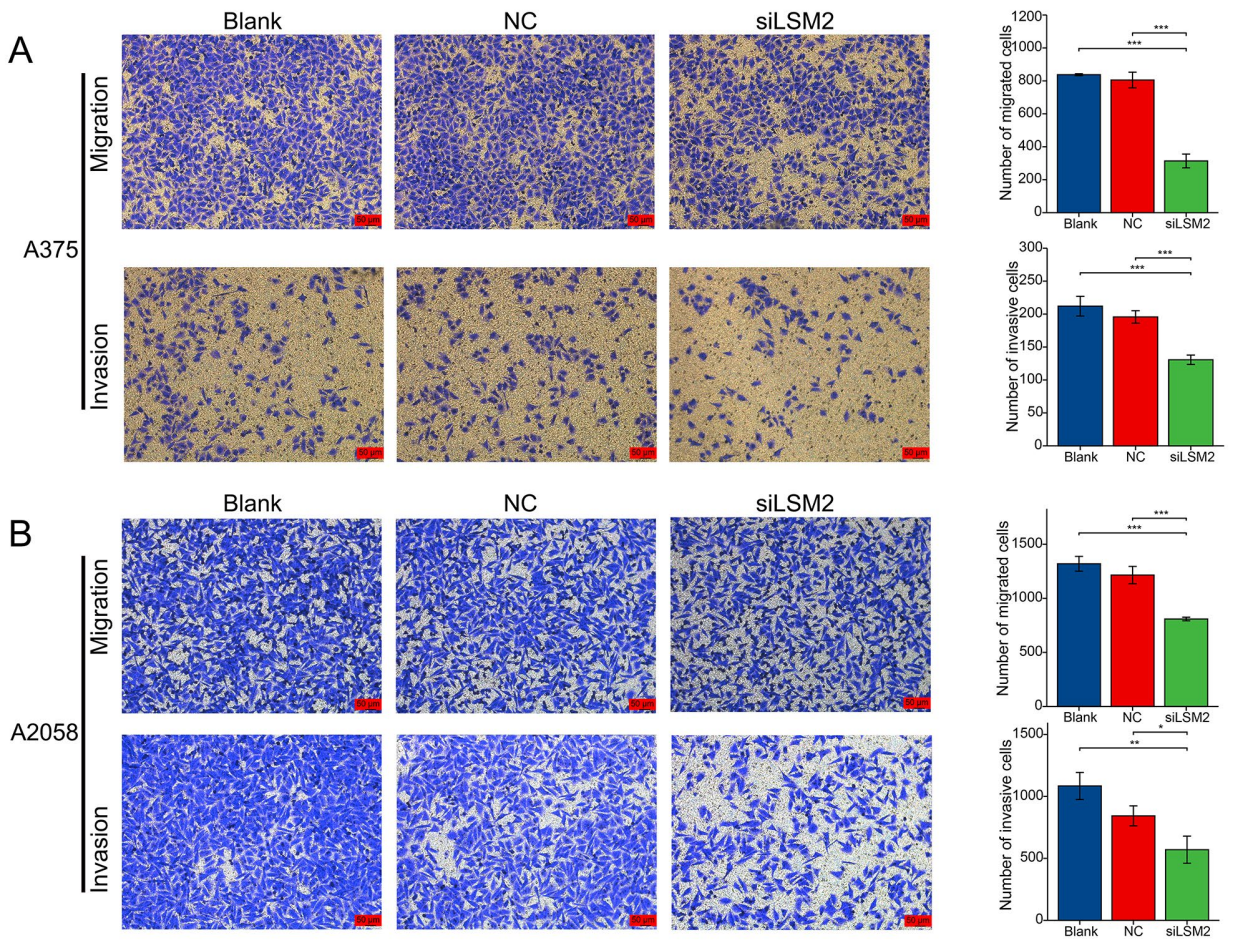
Knockdown of LSM2 inhibits the migration and invasion of SKCM cells **A, B** Migration and Invasion analysis of the A375 and A2058 cell lines using Transwell assays

To explore the biological function of LSM2 in SKCM, we downregulated LSM2 in the A375 and A2058 cell lines using siRNA-3 (siLSM2). The CCK-8 assay revealed that the downregulation of LSM2 significantly decreased cell number. Further, significant differences were found between the two cell lines and the siLSM2 after 24 h (A375, *p* < 0.01; A2058, *p* < 0.001), 48 h (A375, *p* < 0.001; A2058, *p* < 0.001), and 72 h (A375, *p* < 0.001; A2058, *p* < 0.001) (Fig. 7A, C). The colony formation assay revealed that LSM2 silencing decreased the proliferation ability of A375 and A2058 cells (Fig. 7B, D A375, p < 0.05; A2058, *p* < 0.001). We further assessed the invasive and metastatic abilities of these cells, which were markedly decreased after LSM2 knockdown in vitro. Significant differences in cell migration were observed in the wound-healing assays (*p* < 0.05, Fig. 8A, B). A375 and A2058 cells were incubated with siLSM2 for 24 h. A significant reduction in the scratched areas was found in the NC group compared with the siLSM2 group (Fig. 8A, B). Both A375-siLSM2 (migration assay, *p* < 0.001; invasion assay, *p* < 0.001) and A2058-siLSM2 (migration assay, *p* < 0.05; invasion assay, *p* < 0.001) displayed less efficient migration to the lower chamber than the NC controls (Fig. 9A, B).

## Discussion

High-throughput techniques have proven useful for screening potential biomarkers for tumors [27, 28]. In this study, bioinformatic analysis was used to assess the oncogenes associated with SKCM. In TCGA and GEO datasets, LSM2 mRNA was overexpressed in patients with SKCM. In addition, in the BioGPS database, LSM2 was found to be highly expressed in cutaneous melanoma cells compared to normal skin cells. Markedly upregulated LSM2 mRNA expression was also found in breast cancer (BRCA) [29], pancreatic ductal adenocarcinoma (PDAC) [30], and hepatocellular carcinoma (HCC) [31]. Germline variants and somatic mutations of LSM2, which belong to mRNA splicing-related genes, have been confirmed to be high-risk factors for lung cancer [21, 32]. LSM2 has been identified as a potential functional gene of major histocompatibility complex (MHC) III [33]. According to prior studies, genetic variants in the MHC III regions are related to BRCA [34]. These results highlight the oncogenic potential of LSM2. In TCGA database, the OS and PFS of patients with high LSM2 mRNA expression were significantly shorter than those of patients with low LSM2 mRNA expression. A time-dependent ROC curve confirmed the specificity and sensitivity of the prognostic value of LSM2, aligning with our previous study [22], in which the prognostic value of LSM2 mRNA in patients with SKCM was assessed using GEPIA. Based on prior results, high LSM2 expression is associated with poor survival. Therefore, LSM2 mRNA level was identified as an independent risk factor for shorter OS and PFS in SKCM. Similarly, some previous studies revealed that high LSM2 mRNA expression was significantly associated with poor OS in patients with BRCA [29] and HCC [31]. Thus, LSM2 may be an oncogene. Oncogenes are key genes that promote the transformation of normal cells into malignant cells, whereas tumor-suppressive genes inhibit the development of cancer. The somatic gain-of-function of oncogenes or loss-of-function of tumor-suppressive genes significantly affects the occurrence and development of tumors [35, 36].

In most prior studies, LSM2 expression was found to be limited to the mRNA level. Gene expression is well known as a complex process that is regulated at different levels, including transcription [37], mRNA processing [38], protein translation [39, 40], and post-translation [41]. From a technical perspective, both transcriptomics and proteomics are mature technologies that can provide reliable and comprehensive quantitative data. In the process of gene expression, mRNA is closer to the genome and more directly reflects upstream processes, such as transcription factor activity, RNA processing events, and epigenetic regulation. Unlike transcriptomics, proteomics probes gene expression phases that are closer to what individuals consider as “gene function” and is thus more directly related to phenotypes [42]. On one hand, the protein level is more robust for assessing functionally unrelated mRNA level variability. On the other hand, post-transcriptional and post-translational regulation induce important functional changes in protein abundance, which cannot be observed at the mRNA level. To better understand how the genome impacts the phenotype, the mRNA and protein levels of the gene must be determined.

LSM2 protein expression in SKCM and normal skin tissues collected from our hospital was determined via IHC. LSM2 was found to be upregulated in SKCM and was mainly located in the nucleus and cytoplasm of cells. This result aligns with that from the Human Protein Atlas (HPA, https://www.proteinatlas.org/). Another study verified the overexpression of LSM2 in nasopharyngeal carcinoma (NPC) tissues using IHC [43]. Further analysis of the associations between LSM2 protein expression and the clinical features of the 44 patients with SKCM in clinical cohort revealed that high LSM2 protein expression was positively associated with melanoma ulcer, advanced TNM stage, high Clark level, and deep Breslow depth, indicating the oncogenic characteristics of LSM2 in SKCM. Similarly, a previous study revealed that LSM2 is correlated with increasing tumor stage in BRCA [29]. The prognostic value of LSM2 protein expression was explored using a KM plot, and high LSM2 protein expression was found to be related to poor prognosis and recurrence of patients with SKCM at the clinical cohort. Univariate analysis revealed that high LSM2 expression, pathological stage, TNM stage, Clark level, and Breslow depth were independent prognostic factors for patients with SKCM, while multivariate analysis demonstrated that pathological stage, Clark level, and Breslow depth were independent prognostic factors. This difference may be attributed to the small sample size of this study. The depth of infiltration and vertical tumor thickness are the most important prognostic factors for patients with SKCM. Clark level and Breslow depth are two staging systems that are available for the evaluation of depth [44, 45].

To clarify the role of LSM2 in SKCM, cell function analysis was performed. The DepMap database, which includes CRISPR-Cas9 and RNAi datasets, is helpful for determining the essential gene for cell growth and survival. This database provides an effective and simple method for predicting and defining genes necessary for cell viability [46]. In this study, using knockout or knockdown techniques, most LSM2 dependency scores of the SKCM cell lines were < -0.5, indicating that knocking out or knocking down LSM2 mRNA could significantly affect the growth of cutaneous melanoma cell lines in the DepMap database. Subsequently, in vitro experiments revealed that LSM2 silencing slowed down the growth and cloning of SKCM cells and decreased their migration and invasion abilities. A study revealed that non-small-cell lung cancer (NSCLC) cell lines are particularly sensitive to the loss of the LSM2-8 protein complex (especially LSM2, LSM4, and LSM5) [47]. Another study performed using human HeLa cells revealed that a decrease in the level of LSM2 can result in alterations in the alternative splicing patterns of genes involved in cell proliferation and/or apoptosis [48]. SNRPC, CSNK2B, and ZNRD1 are significantly associated with LSM2 in SKCM development and progression [22]. Loss of function and gain assays indicated that SNRPC can promote epithelial–mesenchymal transition (EMT) and the motility of HCC cells in vitro [49]. In vitro and in vivo experiments on gastric cancer (GC) concluded that CSNK2B promotes the proliferation and migration of GC cells [50]. Knockdown of ZNRD1 could inhibit the proliferation, colony formation, invasion, and migration of HCC cells [51]. These genes may act with LSM2 in SKCM malignancies.

This study had some limitations. First, owing to the small sample size of the specimens and corresponding clinical data collected at our hospital, bias could not be ruled out. Larger SKCM cohorts and more prospective data are needed to explore LSM2 expression and its relationship with clinical features. Second, further in vivo experiments should be conducted to explore the underlying role of LSM2 in SKCM patients. Third, LSM2 was revealed to inhibit SKCM cell proliferation, migration, and invasion; however, further biological functional experiments must be performed to elucidate the detailed mechanism.

## Conclusions

In general, LSM2 was found to be overexpressed and associated with poor prognosis in patients with SKCM at the mRNA and protein levels. Upregulated LSM2 was positively correlated with melanoma ulcers, advanced TNM stage, high Clark level, and deep Breslow depth. Silencing of LSM2 inhibited SKCM cell growth, colony formation, migration, and invasion. LSM2 may be a novel biomarker for prognosis and a potential therapeutic target in patients with SKCM.

## Electronic supplementary material

Below is the link to the electronic supplementary material.

**Figure S1**: Film 1 and 2 were exposed at the same time. The two films were used to present western blot (WB) results of LSM2 and GAPDH in A375 cells. The red labeled bands are the WB bands of Figure 6B in this paper.

**Figure S2**: Film 1, 2, and 3 were exposed at the same time. The three films were used to show western blot (WB) results of LSM2 and GAPDH in A2058 cells. The red labeled bands are the WB bands of Figure 6C in this paper.

## Data Availability

The data in this study can be obtained from the corresponding author upon request.

## Abbreviations

CCK8: Cell counting kit-8
CGI: Cytosine phosphate guanosine island
CI: Confidence interval
CCLE: Cancer cell line encyclopedia
DFS: Disease-free survival
DepMap: Cancer Dependency Map
EMT: Epithelial mesenchymal transition
GC: Gastric cancer
HR: Hazard ratio
HE: Hematoxylin and eosin
HCC: Hepatocellular carcinoma
IHC: Immunohistochemistry
KM: Kaplan-Meier
LSM: Sm-like MHC:Major histocompatibility complex
NCBI: the National Center for Biotechnology Information
NPC: Nasopharyngeal carcinoma
NSCLC: Non-small-cell lung cancer
OV: Ovarian cancer
OS: Overall survival
PFS: Progression-free survival
PDAC: Pancreatic ductal adenocarcinoma
ROC: Receiver operator characteristic
RT-PCR: Real-time polymerase
SKCM: Skin cutaneous melanoma
siRNA: Small interfering RNA
TNM: Tumor-nodule-metastasis
TMA: Tissue microarray
