# Supplementary material for: LSM2 is associated with a poor prognosis and promotes cell proliferation, migration, and invasion in skin cutaneous melanoma
Source: BMC Med Genomics. 2023 Jun 13;16:129. doi: 10.1186/s12920-023-01564-1 (PMC10262536; doi:10.1186/s12920-023-01564-1)
Supplement: Supplementary file 1 — Figure S1: Film 1 and 2 were exposed at the same time. The two films were used to present western blot (WB) results of LSM2 and GAPDH in A375 cells. The red labeled bands are the WB bands of Figure 6B in this paper. [file 12920_2023_1564_MOESM1_ESM.pdf]

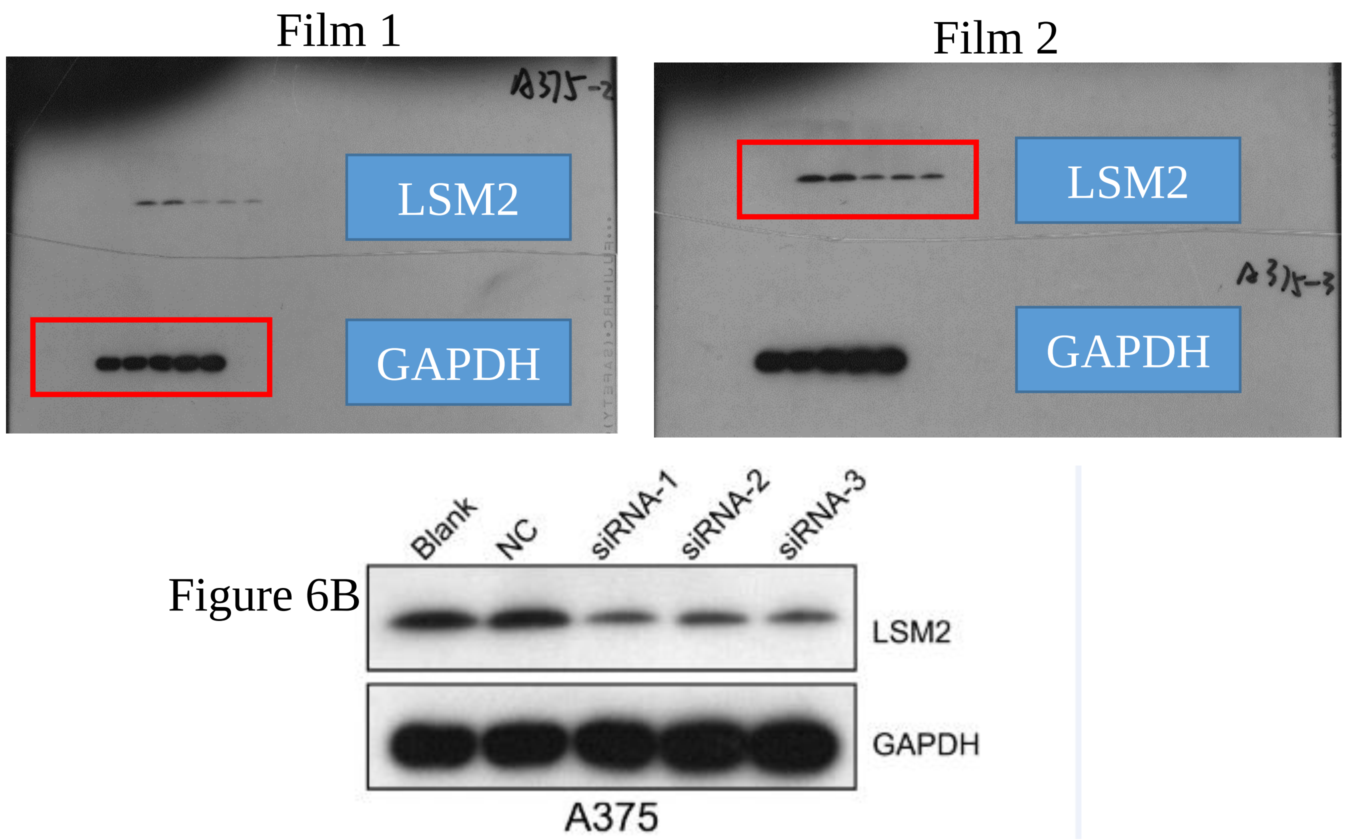

Supplementary Figure S1

Film 1 and 2 were exposed at the same time. The two films were used to present western blot (WB) results of LSM2 and GAPDH in A375 cells. The red labeled bands are the WB bands of Figure 6B in this paper.
