# Supplementary material for: LSM2 is associated with a poor prognosis and promotes cell proliferation, migration, and invasion in skin cutaneous melanoma
Source: BMC Med Genomics. 2023 Jun 13;16:129. doi: 10.1186/s12920-023-01564-1 (PMC10262536; doi:10.1186/s12920-023-01564-1)
Supplement: Supplementary file 2 — Figure S2: Film 1, 2, and 3 were exposed at the same time. The three films were used to show western blot (WB) results of LSM2 and GAPDH in A2058 cells. The red labeled bands are the WB bands of Figure 6C in this paper. [file 12920_2023_1564_MOESM2_ESM.pdf]

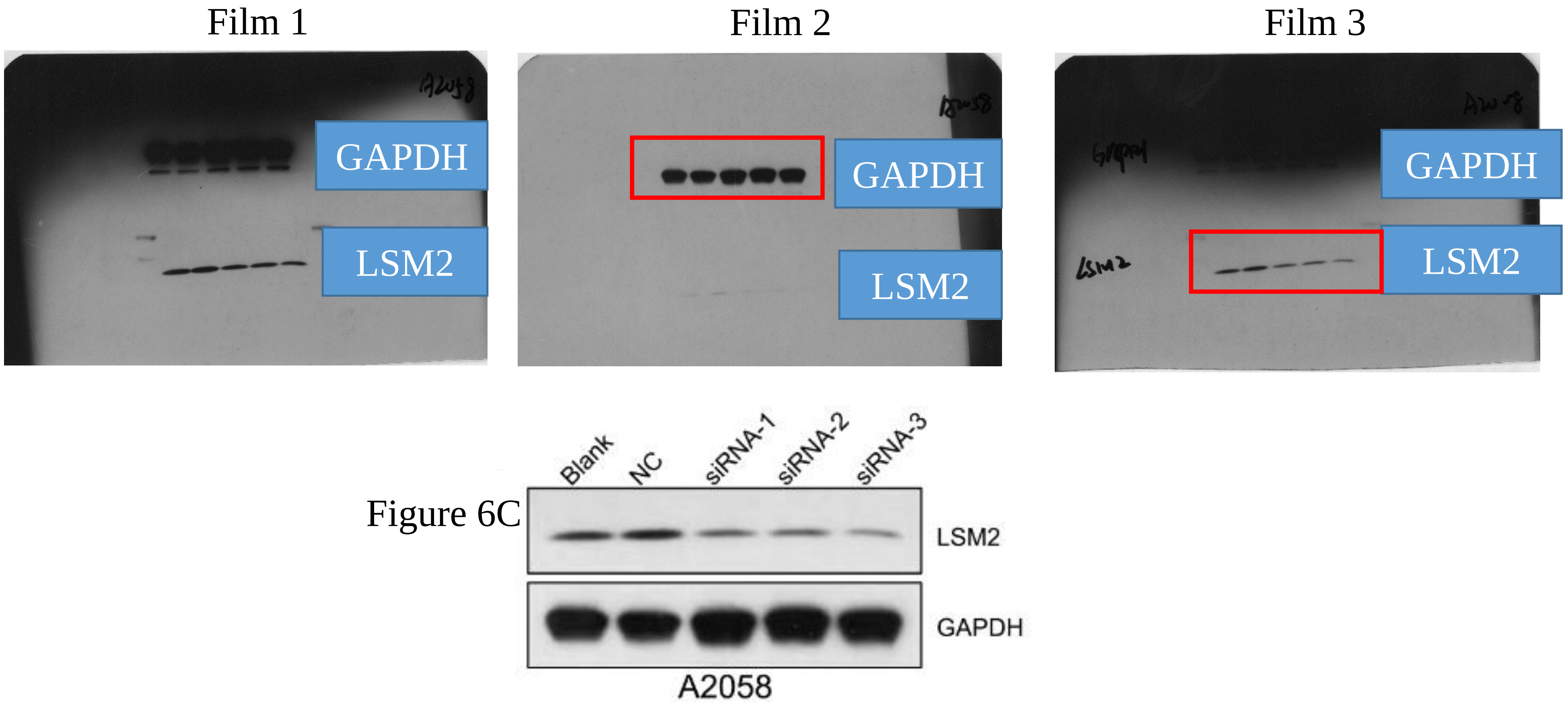

Supplementary Figure S2

Film 1, 2, and 3 were exposed at the same time. The three films were used to show western blot (WB) results of LSM2 and GAPDH in A2058 cells. The red labeled bands are the WB bands of Figure 6C in this paper.
